# Sickness certificates in Sweden: did the new guidelines improve their quality?

**DOI:** 10.1186/1471-2458-12-907

**Published:** 2012-10-26

**Authors:** Emma Nilsing, Elsy Söderberg, Birgitta Öberg

**Affiliations:** 1 Department of Medical and Health Sciences, Division of Physiotherapy, Linköping University, Linköping, Sweden; 2 Department of Medical and Health Sciences, Division of Community Medicine, Linköping University, Linköping, Sweden

**Keywords:** Sickness certificates, International Classification of Functioning, Disability and Health (ICF), Rehabilitation, Sick leave, Physicians, Functioning, Work ability, Sweden

## Abstract

**Background:**

Long-term sickness absence is high in many Western countries. In Sweden and many other countries, decisions on entitlement to sickness benefits and return to work measures are based on information provided by physicians in sickness certificates. The quality demands, as stressed by the Swedish sick leave guidelines from 2008, included accurate sickness certificates with assessment of functioning clearly documented. This study aims to compare quality of sickness certificates between 2007 and 2009 in Östergötland County, Sweden. Quality is defined in terms of descriptions of functioning with the use of activity and participation according to WHO’s International Classification of Functioning, Disability and Health (ICF), and in prescriptions of early rehabilitation.

**Methods:**

During two weeks in 2007 and four weeks in 2009, all certificates had been collected upon arrival to the social insurance office in Östergötland County, Sweden. Four hundred seventy-five new certificates were included in 2007 and 501 in 2009. Prolongations of sick leave were included until the last date of sick listing. Free text on functioning was analysed deductively using the ICF framework, and placed into categories (body functions/structures, activity, participation, no description) for statistical analysis.

**Results:**

The majority of the certificates were issued for musculoskeletal diseases or mental disorders. Text on functioning could be classified into the components of ICF in 65% and 78% of sickness certificates issued in 2007 and 2009, respectively. Descriptions according to body components such as “sensations of pain” or “emotional functions” were given in 58% of the certificates from 2007 and in 65% from 2009. The activity component, for example “walking” or “handling stress”, was more frequent in certificates issued in 2009 compared with 2007 (33% versus 26%). Prescriptions of early rehabilitation increased from 27% in 2007 to 35% in 2009, primarily due to more counseling.

**Conclusions:**

An improvement of the quality between certificates collected in 2007 and 2009 was demonstrated in Östergötland County, Sweden. The certificates from 2009 provided more information linkable to ICF and incorporated an increased use of activity limitations when describing patients’ functioning. Still, activity limitations and prescriptions of early rehabilitation were only present in one-third of the sickness certificates.

## Background

Long-term sickness absence due to work disability is high in many Western countries, especially in Sweden, Finland and the Netherlands [1-3]. In 2011, 8% of the Swedish working-age population received sickness benefits and 7% received incapacity benefits [4]. Even though social security system differs between countries, medical assessments of work disability are similar [5]. Many Western countries have two prerequisites for entitlement to sickness benefits: namely, the individual must have a disease or injury and this disease or injury must impair the ability to work [2,5]. The assessment of work ability is made by physicians, and in most countries the information should be provided in a sickness certificate [3]. In Sweden, after seven days of self-certification, the assessment is made by physicians in their role as medical expert for another authority, such as the social insurance office, and the information is given in a sickness certificate. The formal decision on entitlement to sickness benefits is then made by the social insurance office, based on the information given in the certificate and the patient’s own request for sick leave [2]. Sickness certificates are thus an important means for communication between health care providers and the social insurance office. However, information provided in sickness certificates is often too scarce to allow for a firm decision regarding entitlement to sickness benefits [6] or to detect cases where modified work conditions may reduce the length of sick leave [7]. Assessments of functioning and work ability are challenging tasks for physicians [8-13], and the assessments and the sickness certification process are little supported with evidence or instruments [14]. One way to improve the quality and transparency of the work ability assessments is to use guidelines, but guidelines for sickness certifications and disability benefits are thus far scarce [3,14,15].

In 2008, governmental decisions including time-limits for the review of eligibility and maximum length of sick leave within the rehabilitation chain, sick leave guidelines and a new sickness certificate were implemented in Sweden [16]. By the time of the implementation, political discussions, public debates and the focus in media concerned the time-limits for sick leave length and the implementation of the guidelines. The guidelines aim not only to facilitate the management of sick leave cases, but also to provide a structure for collaboration between health care sector and social security system, and to facilitate encounters with patients. Emphasis is put on the notion that certifying sick leave is an active intervention requiring the same high quality standards as other health care activities. The guidelines comprise general principles regarding the management of sick leave [16] and specific recommendations for sick leave, length, and grade according to diagnoses [17]. The general principles include recommendations regarding required documentation in sickness certificates, the assessment of work ability as a tool for intervention, patient participation, early commitment, contact with the work place, assessment of functioning, and assessment of work ability related to work demands and possible work modifications [16]. The purpose of the specific recommendations is to support the physician in performing these tasks and to communicate with the patient and other stakeholders, by giving timelines for recovery, interventions, and sick leave length corresponding to specific diagnoses and work categories [17]. These specific guidelines were primarily based on consensus discussions among medical experts within different specialities’ [16]. The quality demands include accurate sickness certificates with assessments of functioning and work ability clearly documented [16]. The patient’s functioning should explicitly be expressed in terms of what the patient is expected to be capable or not capable of performing at the workplace [16]. This is a change in the perspective, from disease and symptoms towards activity and participation, in line with the World Health Organisation’s (WHO) International Classification of Functioning, Disability, and Health (ICF) [18].

The guidelines were disseminated with different approaches emphasizing information and education but also including financial incitements for the health care to perform the implementation [19]. The strategies for the implementation involved the actors, the health care sector and social security system, which were responsible for leaderships and systems regarding professional competence, quality improvements and evaluations of the sick leave process at their local levels. The implementation strategies in Östergötland County focused on information and education to managers and those employees involved in sickness certifications. The strategies included a sickness absence committee with representatives from the health care sector and social security system; education interventions; seminars and discussions with primarily physicians; web pages and booklets with information directed at physicians and patients. Similar activities were performed in other County Councils [19]. A recently published cross-sectional study showed that one year after the implementation of the Swedish sick leave guidelines, a majority of the general practitioners reported to use these guidelines and considered them useful primarily in contacts with patients [20]. To the best of our knowledge, studies investigating the effect of the guidelines regarding sickness certification in Sweden have so far not been published. The outermost aim of implementing guidelines is to improve patient care, but it is shown to be a slow and unpredictable process [21,22], and there are many potential factors that may influence the change process [22]. The following factors, also named determinants, have shown to re-emerge in several theories and frameworks: the characteristics of the implementation object; the strategies for the implementation; the internal and external context; and the characteristics of the target group [22-25]. These determinants and their underlying theories will comprise the framework for this study.

In the development of the guidelines, an important question was how the assessment of work ability by physicians can be improved. The ICF framework may help the physician to describe the work disability as bio psychosocial phenomena instead of a biomedical [18,26]. There is though a discussion about the limitations of the ICF in work ability assessments, such as the lack of classification of personal factors [27], a causal relationship between the components, and for not capturing the work characteristics sufficiently [26]. Despite that, ICF core sets for functional assessments in disability benefit claims have been developed, which may facilitate the description of functional capacity and enable comparisons in European social security systems [28]. In two previous studies, it was found that ICF could be used to structure information given in sickness certificates, complementing the diagnosis classified according to the WHO’s International Classification of Diseases (ICD-10) and clarifying the assessment of functioning and work ability [29,30]. The use of ICF to structure information provided in sickness certificates may be one way to improve the quality of work ability assessments and sickness certificates by shifting the focus from body impairments towards activity and participation.

This study aims to compare quality of sickness certificates between 2007 and 2009 in Östergötland County, Sweden. Quality is defined in terms of descriptions of functioning with the use of activity and participation according to ICF, and in prescriptions of early rehabilitation. We hypothesized that the quality would improve between the certificates collected in 2007 and those collected in 2009. The results from the study may be used to develop strategies for enhancing quality in sickness certificates, which could improve the basis for decisions on entitlement to sickness benefits.

## Methods

This is a comparative study using sickness certificates issued for a new sick leave period. Data was collected in 2007 and in 2009, before and after implementation of Swedish sick leave guidelines [16].

All new sickness certificates arriving to the social insurance office in Östergötland county, Sweden (pop. 420,809), were collected consecutively during two weeks (weeks 40-41) in 2007 (n = 497) and four weeks (weeks 42-45) in 2009 (n = 508). In addition, any incoming certificate that prolonged sick leave was collected until the current sick leave period ended. In the sample from 2007, 22 certificates were excluded because of not certifying a new sick leave period (n = 16), death (n = 4), or incorrect personal identity number (n = 2). In 2009, seven certificates were excluded because of death (n = 6) or having an infectious disease monitored by the Communicable Diseases Act (n = 1). Four hundred seventy-five and 501 new sickness certificates were included in the analysis for 2007 and 2009, respectively. In total, 1,311 certificates were issued for the sample in 2007 and 1,201 for 2009.

Information collected from the first sickness certificate included the following aspects: affiliation of the certifying physician (primary health care (PHC), occupational health service (OHS), hospital, and private clinic), patient’s age (≤ 24, 25-34, 35-44, 45-54, or ≥ 55 years), patient’s sex, main disease resulting in sick leave according to ICD-10 codes, and description of functioning classified according to ICF. The certificates do not provide information on physician characteristics. Information on sick leave length, prescribed interventions and return-to-work (RTW) measures was collected from the total collection of certificates. The ICD-10 codes were categorized as follows: Mental and behavior disorders (F00-F99) into mental disorders (MD), Diseases of the musculoskeletal system and connective tissue (M00-M99) into musculoskeletal diseases (MSD), Diseases of the circulatory system (I00-I99) and Diseases of the respiratory system (J00-J99) into circulatory and respiratory diseases (CR), and the remaining codes (A-E, G, H, K-N, O-Z) into the group “other diagnoses”. Similar categorization has been used previously [31]. Sick leave length was defined as the number of sick leave days in current spell during the total study [32]. Information on sick leave length per patient was obtained by calculating the number of sick leave days prescribed in the first sickness certificate plus additional prolonging certificates. Days of partial absence due to sickness were combined (e.g., two days of 50% sick leave were counted as one day) [33].

Text written in the certificates in response to anamnesis, clinical findings, and the question “how does the disease limit the patient’s ability/activity” was analysed using content analysis [34,35] with ICF as a theoretical framework [18]. In the certificate that was used in the sample from 2009, the following in italics was added to the certificate from 2007: “clinical findings *on organ level (impairment)*” and “how does the disease limit the patient’s ability/activity *on an individual level (activity limitation)*”. The text was read and meaningful concepts were identified and classified into the different components of ICF: body functions/structures, activities, and participation [18,36]. Table 1 gives an overview of the analysis. Insufficient text, such as “operated” or no information given at all, was assigned to a separate category “no description” [36]. For certificates issued in 2007, the analysis was performed by two independent researchers and followed by a consensus meeting between the two researchers and an adjudicator, as described in detail previously [29]. The analysis was then assumed to be reliable and carried out in the same way for certificates issued in 2009 by one researcher (EN). Concepts difficult to link into the most precise category were discussed in consensus meetings (EN and BÖ). Text regarding functioning was placed into categories (body functions/structures, activity, participation, no description) for statistical analysis [34,35].

**Table 1:** Overview of the analysis with examples of quotations classified into the different components of ICF

**Quotation**	**Meaning unit**	**Body**	**Activity**	**Participation**	**No description**
Anxiety, difficulties to concentrate and sleep, which generates tiredness daytime.	Anxiety, concentration, sleep disturbance, tired	b152 Emotional functions, b140 Attention functions, b134 Sleep functions, b130 Energy and drive functions			
Patient has difficult to sit for a long time. Work as a driver. Cannot load in or out of the car because of pain and stiffness.	Prolonged sitting, works as a driver, loading, pain, stiffness	b280 Sensation of pain, b780 Sensations related to muscles and movement functions	d4103 Maintaining a sitting position, d430 Lifting and carrying objects	d850 Remunerative employment	
Because of the side effects of the treatment not able to work.					Neither the side effects or the effect on functioning are described

The prescription “intervention essential for recovery of ability” included free text, which was categorized as rehabilitation, medical intervention, or no intervention. Rehabilitation comprised physiotherapy, counseling (i.e. therapy conversation), occupational therapy, or referral to a rehabilitation clinic or OHS. In this study, rehabilitation prescribed in the first certificate, or within 28 days of sick leave, was defined as early rehabilitation. The cut-off point of less than 28 days of sick leave was chosen based on the common division of patients with back pain: acute (< 4 weeks), sub-acute (4-12 weeks), and chronic pain (> 12 weeks) [37]. Prescriptions of medication or advice were defined as medical interventions, and no intervention refers to certificates without a prescription of any intervention. In the certificate the physician should respond to the question “Are return to work measures needed?” by choosing one of the four alternatives: yes, no, cannot be assessed now, or in need for OHS. This information is important for the employer and the social insurance office in order to coordinate return to work (RTW) measures. The answers “no” and “cannot be assessed now” were categorized into “no”, and “yes” and “in need for OHS” into “yes”.

Pearson’s chi-squared tests were used to analyse associations between categorical/nominal variables. Student’s t-test was used for group comparisons of continuous variables. Mean, median, and 95% confidence intervals were calculated for measures of sick leave. Differences were considered significant at *p* < 0.05.

The Research Ethics Committee of the Faculty of Health Sciences of Linköping University, Sweden approved the study.

## Results

As shown in Table 2, there were no significant differences between the samples in descriptive data (age, sex, distribution of diagnostic group, or affiliation of certifying physician), except for sick leave length. The majority of the certificates were issued for patients with MSD or MD. The proportion of certificates issued for MD increased in 2009 compared with those issued in 2007, but not significantly. “Other diagnoses” consist of a wide range of diagnoses; greatest among them were injuries (S00-T98, about 10%). Physicians at hospitals and in PHC primarily issued certificates. In 2007, there were more new sick leave spells and the sick leave length was longer than in 2009. The median prescribed sick leave length was 36 days in 2007 and 32 days in 2009. The sick leave length exceeded 365 days in 33 certificates in 2007 and in 15 certificates in 2009, where a maximum timeline of sick leave length was set according to legislation changes.

**Table 2:** Descriptive information provided in sickness certificates issued in 2007 and 2009

**Variable**	**2007N= 475**		**2009N= 501**		**P-value**
	**%**	**n**	**%**	**n**	
**Sex**					
Man	38	182	34	171	
Woman	62	293	66	330	0.174
Total	100	475	100	501	
**Age average**					
mean, SD	45	12	45	12	0.566
**Age interval**					
≤24	6	26	7	33	
25-34	19	90	17	86	
35-44	23	108	23	116	
45-54	24	112	25	125	
≥55	29	139	28	141	0.871
Total	100	475	100	501	
**Diagnostic group**					
MD	17	80	21	105	
MSD	29	137	28	138	
CR	9	42	11	55	
Other	45	211	40	196	0.166
Total	99	470	99	494	
**Physician affiliation**					
PHC	43	201	42	210	
OHS	5	24	4	19	
Private	8	39	10	51	
Hospital	44	206	44	219	0.591
Total	99	470	100	499	
**Total sick leave length**					
Median, CI	36	82-107	32	57-72	
Mean, SD	94	139	65	87	<0.001

MD, mental disorders; MSD musculoskeletal diseases; CR, circulatory and respiratory diseases; Other, other diagnostic groups; PHC, primary health care; OHS, occupational health services.

CI, confidence interval 95%.

### Description of functioning

Based on the question “how does the disease limit the patient’s ability/activity”, 35% of certificates in 2007 did not provide enough information for classification according to ICF. This lack of information decreased to 21% in 2009. When functioning could be classified into ICF it was mainly provided on the component of the body. Table 3 presents the ICF components provided in all parts of the certificate. In the answer to the question “how does the disease limit the patient’s ability/activity”, the proportion of components provided for body and activity had increased significantly in 2009. The certificates issued in 2009 reported more frequently the patient’s type of work compared with those issued in 2007 (95 and 78%, respectively).

**Table 3:** Descriptions of functioning in different parts of sickness certificates and classified into ICF components

**Functioning**	**2007N= 475**		**2009N= 501**		**P-value**
	**%**	**n**	**%**	**n**	
**Anamnesis**					
Body	82	387	80	402	0.624
Activity	15	73	12	62	0.176
Participation	7	33	4	19	0.028
**Clinical findings**					
Body	75	355	75	376	0.910
Activity	6	26	5	26	0.843
Participation	0.6	3	0.2	1	0.361ª
**How the disease limits ability/activity**					
Body	58	276	65	325	0.03
Activity	26	125	33	164	0.028
Participation	6.5	31	8	39	0.446
All parts*					
Body	92	438	94	472	0.213
Activity	35	168	39	197	0.202
Participation	12	59	11	56	0.547
**Information on work**					
Yes	78	370	95	477	<0.001

Column sum is more than 100% because some certificates provided information on functioning classified into more than one component.

ª Fisher’s exact test, 2-sided.* All parts, information on functioning collected from anamnesis, clinical findings and the question “how does the disease limit the patient’s ability/activity”.

### Prescription of intervention

There were differences between the samples in prescribed interventions early in the sick leave, but not during the total sick leave period, with the exception of RTW measures (Table 4). Prescription of medicine was the most common intervention. Advice on activity was more common in 2009 compared with 2007. Prescriptions of early rehabilitation were more frequent in certificates issued in 2009 than in 2007 (35% versus 27%), mainly due to more counseling. Information on need for RTW measures was less frequent in certificates issued in 2009 than in 2007 (8% and 13%, respectively). Information on interventions in the total sick leave period was lacking in 26% of the certificates issued in 2007 and 22% in 2009.

**Table 4:** Interventions prescribed during the sick leave in sickness certificates issued in 2007 and 2009

**Intervention**	**2007N= 475**		**2009N= 501**		**P-value**
	**%**	**n**	**%**	**n**	
**Early in the sick leave**					
Medicine/advice	56	264	58	290	0.467
Medicine	43	205	43	215	0.939
Advice on rest	10	49	13	67	0.140
Advice on activity	3	14	6	30	0.022
Rehabilitation	27	130	35	174	0.013
Physiotherapy	19	92	19	97	0.998
Counselling	8	38	15	74	0.001
Occupational therapy	1	4	1	5	1.00ª
**In the total sick leave**					
Medicine/advice	62	296	62	310	0.888
Medicine	50	235	46	232	0.322
Advice on rest	11	53	15	73	0.112
Advice on activity	6	26	7	35	0.329
Rehabilitation	35	167	39	193	0.276
Physiotherapy	24	112	23	113	0.704
Counselling	12	59	16	78	0.157
Occupational therapy	2	10	2	8	0.555
**RTW measures needed**					
Yes	13	61	8	41	0.017

Sum of sub grouped interventions may not be equal to medicine/advice or rehabilitation since some certificates provided information on more than one intervention (for example both counseling and physiotherapy). Early in the sick leave means that information is collected from the first certificates or from certificates issued within 28 days of sick leave. ª Fisher’s exact test, 2-sided. RTW, return to work.

## Discussion

We found that sickness certificates issued in 2009 provided more information on patients’ functioning than those issued in 2007 (78% versus 65%, respectively). Descriptions of functioning according to activity and prescriptions of early rehabilitation increased from approximately one-fourth of certificates in 2007 to one-third in 2009. However, even after implementation of sick leave guidelines, body impairments still dominate the descriptions of functioning in sickness certificates. These results do not comply with recommendations of the Swedish sick leave guidelines, which emphasize that assessments of functioning should be expressed in terms of what the individual is expected to be capable versus not capable to perform [16]. This correlates with the activity or participation components of ICF [18]. In addition, we found that the proportions of descriptions of functioning classified into ICF contrasted with the suggested core sets for disability evaluations in social security, where 15 out of 20 core sets were from activity and participation and only five from body functions [28]. The scarce use of activity and participation in the present study may be attributed to several factors such as insufficient adherence to the guidelines in practice or to the challenges many physicians face in assessing patients’ functioning and work ability [8-13]. The focus on body impairments may also be explained by the legislation, since the Swedish Social Insurance Act requires that the work ability is impaired due to a disease or injury, and does not accept social, economic or labour market related reasons for granting sickness benefits [2]. This distinction between medical and other reasons may be difficult in practice [10,12] and might strengthen the biomedical way of describing functioning in sickness certificates. Furthermore, physicians may have insufficient knowledge about the ICF framework to use it as guidance when issuing sickness certificates. Prescriptions of early rehabilitation increased as well in 2009, mainly due to more counseling. The frequency of prescriptions of early rehabilitation and RTW measures appears though to be low, indicating that early interventions and contact with work may not be performed as recommended by the guidelines [16]. The timing of assessment of work ability as well as tailored interventions during sick leave are important for RTW [38], especially since administrative and treatment delays predict long-term work disability [39].

In a recent survey, the majority of the general practitioners reported to use the Swedish sick leave guidelines and found them useful in several aspects [20]. However, if our results reflect actual clinical practice, there may be a gap between sick leave guidelines and the practice of issuing sickness certificates. In general, insufficient adherence to guidelines is attributed to characteristics of the guideline, effectiveness of the chosen implementation strategies, and contextual factors that impact the implementation process and outcomes [22]. Theories of behavior modification also offer an explanation as to why practice change is often difficult to achieve. Cognitive and social-cognitive theories posit that behavior is influenced by factors such as self-efficacy, motivation, beliefs, attitudes, and subjective norms concerning the behavior in question [40]. These theories tend to emphasize deliberate, conscious behaviours. In contrast, research on habits suggest that the pervasive effect of habits in everyday behavior is key to understanding the difficulty people experience in changing their behavior [41,42]. Although the Swedish guidelines were implemented using several different approaches, the core component of the strategy was education and information, which may simply have been insufficient to change existing habits, at least in the short-term. Further explanations for the results are related to the other determinants: the implementation object, the contextual factors and the target group. The implementation object, i.e. the guidelines, may be characterized as having a relative advantage and utility since there was a need for more knowledge and skills in handling sickness certification before the implementation [43] and afterwards a majority of the general practitioners in a Swedish study reported usefulness of the guidelines [20]. Due to the lack of scientific evidence in the field of work ability assessments in social security [15], the guidelines were primarily based on consensus discussions with medical experts [16]. The adherence to the guidelines might depend more on the involved physicians’ perceptions of the guideline legitimacy, than if the guidelines were based on an expert opinion or scientific evidence. However, adherence to evidence based guidelines might yield better effects in terms of sick leave length and return-to-work rates [44]. The quality improvement could have been greater, if the target groups’ attitudes and habits in handling sick leave cases had received more attention.

We included all the new sickness certificates that the social insurance office received during the two study periods. The advantage of almost no external or internal loss enables higher validity. In order to achieve the same sample size as collected during two weeks in 2007, four weeks of data collection was needed in 2009. This sample size was deemed to be sufficient for statistical analysis. The two samples were considered comparable since they were collected during the same period of the year and showed no differences in distribution of diagnoses, physician affiliations or other descriptive variables (age, sex). A similar categorization of ICD-10 diagnoses and physician affiliations has been used previously [6,31]. The distribution of diagnoses was representative regarding causes of sick leave [6,45]. Sickness certificates should provide the same type of information regardless affiliation or clinic, and we do not have data on the individual physicians. The differences in sample sizes and sick leave lengths were expected due to the trends of decreasing new sick leave spells and lengths. These differences may also indicate more time for describing functioning according to the quality demands. In this study, length of sick leave was based on sick leave days as a measure of the gathered individual illness burden during the study periods [32]. We combined days of partial sick leave, as previously done [33]. There is though a difference between being on partial or full time sick leave, which especially needs to be considered when analyzing effects of interventions [32]. Strategies for implementing the sick leave guidelines were undertaken at both central and local levels in all County Councils in Sweden [19], and differences at the local level may influence the effect of implementation [22]. Financial incitements were sanctioned to the County Councils and there were no controlling systems for the individual physicians.

Due to the design with two consecutive samples of sickness certificates, a causal relationship between the implementation of the guidelines and the quality improvement is difficult to establish. The guideline implementation was considered the most important change in between the two study periods, but there were also other concomitant factors which may have influenced the findings. First, political discussions, public debates and the focus in media may have increased the awareness of the guidelines and thereby facilitated the implementation. Secondly, the introduction of time-limits for the review of eligibility and maximum length of sick leave within the rehabilitation chain could have influenced the structure of the work ability assessments in the health care sector and social security system. Also the rehabilitation chain may have affected the physicians to write more comprehensive certificates in order to avoid complementary requests or refusals from the social insurance office. Finally, the certificate used in 2009 required explicitly descriptions of functioning according to activity limitations, and when a certified sick leave length exceeds the recommendations in the guidelines, an explanation for this should be stated in the certificate. All of these factors may have to various extents contributed to the increased use of activity limitations when describing functioning and prescribing early rehabilitation in sickness certificates.

The problem with insufficient information in sickness certificates is recognized in different countries such as Sweden, Norway and Slovenia [6,7,31,46]. All results in this study were based on information provided in sickness certificates as an indicator for changes in practice. Since the sickness certificate is a document affecting decisions on entitlement to sickness benefits and RTW measures, the included information is important [2,6,16]. The framework and classification of ICF was used, which enabled a systematic evaluation and description of the data. This limits, however, the evaluation of how functioning is conceptualised in ICF. Only one third of the certificates in 2009 incorporated activity limitations or prescriptions of early rehabilitation, which may question the relevance of the ICF framework for sickness certificates. Previous studies have though shown that ICF complements the diagnostic classification ICD-10 and can be useful in sickness certifications [29,30]. The guidelines as well as the certificate that was introduced in 2008 clearly emphasise information on activity limitations when describing the patient’s functioning. The quality improvement in the sample from 2009 may then partly be explained by the specific request in that certificate. However, activity limitations and participation restrictions were still scarce, which indicate that physicians may not have sufficiently knowledge about ICF to use it adequately. Furthermore, we did not study whether ICF is feasible or contains relevant aspects from a physician perspective. The majority of the certificates provided information that was relative easy to link to ICF and the ICF was therefore considered purposeful for this study. Despite the limitations of ICF in work ability assessments [26,27], ICF creates a common language that is useful in social security [28] and can be used to structure descriptions of functioning and work ability in sickness certificates [29,30]. Future research is needed on development of strategies for enhancing quality in sickness certificates, which could improve the basis for decisions on entitlement to sickness benefits.

## Conclusions

An improvement of the quality between certificates collected in 2007 and 2009 was demonstrated in Östergötland County, Sweden. The certificates from 2009 provided more information linkable to ICF and incorporated an increased use of activity limitations when describing patients’ functioning. Still, activity limitations and prescriptions of early rehabilitation were only present in one-third of the sickness certificates. The implementation of the Swedish sick leave guidelines has probably attributed to the quality improvement, but other factors might also have influenced the results and a causal relationship between the quality improvement and the guidelines is difficult to establish.

## Competing interests

The authors declare that they have no competing interests. All authors are responsible for the content and writing of the paper.

## Authors’ contributions

EN contributed to the design, acquisition of data, analysed and classified the data, and wrote the manuscript. BÖ participated in its design, analysis and classification of the data, and drafting of the manuscript. ES contributed to the design and drafting of the manuscript. All authors read and approved the final manuscript.

## Pre-publication history

The pre-publication history for this paper can be accessed here:

http://www.biomedcentral.com/1471-2458/12/907/prepub
